# Tirzepatide-associated hyponatremia in a patient with known arginine vasopressin deficiency

**DOI:** 10.1210/jcemcr/luag110

**Published:** 2026-04-27

**Authors:** Carmela Caputo

**Affiliations:** Department of Endocrinology and Diabetes, St Vincent's Hospital Melbourne, Fitzroy 3065, Australia; Department of Medicine, The University of Melbourne, Melbourne 3010, Australia

**Keywords:** arginine vasopressin deficiency, desmopressin, hyponatremia, dual glucose-dependent insulinotropic polypeptide and glucagon-like peptide-1 receptor agonists, tirzepatide

## Abstract

This report describes a 53-year-old female individual with known arginine vasopressin deficiency (AVP-D) due to a recurrent Rathke cleft cyst (RCC), with asymptomatic severe hyponatremia (sodium 121 mEq/L [SI: 121 mmol/L]; reference range 135-145 mEq/L [SI: 135-145 mmol/L]) in the setting of several months of tirzepatide use for weight loss. On review of the literature this is the third case of severe hyponatremia (sodium ≤125 mEq/L [SI: ≤125 mmol/L]) reported with the use of tirzepatide and first case in someone with AVP-D. Although hyponatremia associated with tirzepatide is rare, it poses a significant risk for individuals with AVP-D, who are already predisposed to hyponatremia. Clinicians should remain vigilant regarding this potential complication, provide education on desmopressin escape strategies, and carefully consider reductions in desmopressin dosing when co-administering with tirzepatide.

## Introduction

Arginine vasopressin deficiency (AVP-D) is a rare condition affecting approximately 1 in 25 000 people [1]. Hormone replacement of arginine vasopressin (AVP) with desmopressin is effective; however, the complication of dysnatremia, particularly hyponatremia, is common in both outpatient and inpatient settings [2-5].

Drug interactions with desmopressin used in those with AVP-D increase the risk of hyponatremia [6]. Literature highlights an association with the dual glucose-dependent insulinotropic polypeptide (GIP) and glucagon-like peptide 1 (GLP-1) receptor agonist (GIP/GLP-1 RA) tirzepatide and hyponatremia, with 2 recent case reports of severe hyponatremia (sodium ≤125 mEq/L [SI: ≤125 mmol/L]) (reference range 135-145 mEq/L [SI: 135-145 mmol/L]) occurring shortly after starting tirzepatide: these were not involving people with AVP-D [7, 8]. In another case series of 3 people with AVP-D taking GLP-1 RA semaglutide or liraglutide, all 3 noted decreases in urine output requiring marked reductions in their desmopressin dosages [9].

Herein, is a case of severe hyponatremia associated with tirzepatide use in a patient with AVP-D. It raises concern, given the escalating use of tirzepatide and the potential risk in those with AVP-D.

## Case presentation

A 53-year-old postmenopausal woman had a history of recurrent suprasellar Rathke cleft cyst (RCC) and AVP-D. Her initial presentation with the RCC dated back to 1999, necessitating transsphenoidal surgery due to visual impairment. Subsequent recurrences occurred, requiring surgeries in 2003, 2006, and 2011. She developed AVP-D after pituitary surgery in 2006, which resolved following her last surgery in 2011, until the AVP-D symptoms suddenly reappeared in 2015 after a dental procedure. She used various oral desmopressin regimens but most recently, she was on a stable dose of 100 mcg orally 4 times daily. She had a history of hypertension treated with telmisartan 80 mg daily. She did not have any anterior pituitary hormone deficiencies.

In 2016, she was hospitalized with gastroenteritis and found to be severely hyponatremic, with a sodium level of 110 mEq/L [SI: 110 mmol/L] and again in 2024 she developed hyponatremia (sodium 126 mEq/L [SI: 126 mmol/L]) during a COVID-19 infection.

In January 2025, her general practitioner started her on tirzepatide for long-standing weight issues (weight 130 kg). Her weight reduced to 98 kg over 9 months using tirzepatide 10 mg weekly. She was not cognizant of any change to her fluid intake or urine output during this time. In late 2025, she developed mild visual disturbances, prompting her to follow up with her endocrine review. At her last endocrine follow-up in 2022, her pituitary magnetic resonance imaging (MRI) scan was satisfactory with no residual cyst, and her sodium level was 135 mEq/L (SI: 135 mmol/L). She had ongoing care with her general practitioner thereafter and her last sodium was 136 mEq/L (SI: 136 mmol/L) prior to commencing tirzepatide.

## Diagnostic assessment

**Figure 1:**
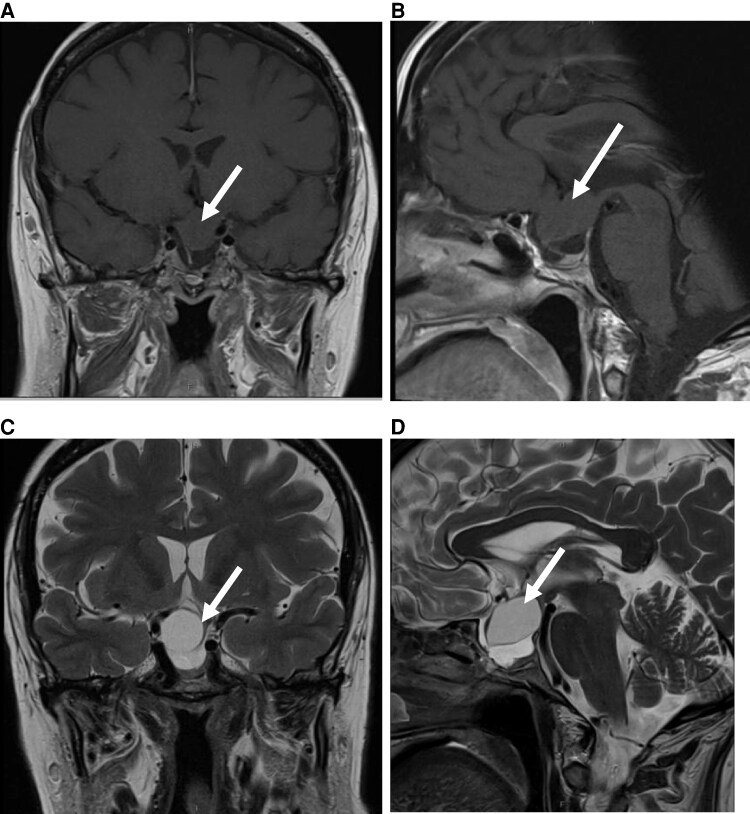
Magnetic imaging resonance scans at current presentation with recurrent Rathke cleft cyst. A-B, A coronal and B sagittal non-contrast T1 showing ovoid-shaped lesion in the suprasellar region measuring 22 × 17 × 18 mm: white arrows. This causes distortion of the pituitary stalk off to the far-right side and displaces the optic chiasm in a superior direction and off to the right of midline. The mass signal on T1 is isointense to brain parenchyma. Beneath this lesion and the pituitary gland which sits at the floor. C-D, C coronal and D sagittal T2 sequence demonstrating the suprasellar cystic lesion.

A pituitary MRI scan was arranged prior to her review, revealing yet another recurrence of the suprasellar cystic lesion measuring 22 × 17 × 18 mm (Fig. 1). She was contacted to arrange hormonal blood tests as well as visual field assessments.

Her visual field evaluation showed a new, right bilateral hemianopsia. Blood tests indicated hyponatremia, with a sodium level of 124 mEq/L [SI: 124 mmol/L]. Her anterior pituitary hormone panel was within normal limits (Table 1).

**Table 1:** Biochemical and hormonal results at presentation

Baseline biochemistry conventional (SI)		Normal range conventional (SI)
Sodium	**124** **mEq/L (124 mmol/L)**	135-145 mEq/L (135-145 mmol/L)
Potassium	4.5 mEq/L (4.5 mmol/L)	3.5- 5.5 mEq/L (3.5-5.5 mmol/L)
Chloride	87 mEq/L (87 mmol/L)	95-110 mEq/L (95-110 mmol/L)
Bicarbonate	24 mEq/L (24 mmol/L)	20-32 mEq/L (20-32 mmol/L)
Urea	4.7 mEq/L (4.7 mmol/L)	3.0-8.0 mEq/L (3.0-8.0 mmol/L)
Creatinine	0.59 mg/dL (52 μmol/L)	0.51-0.96 mg/dL (45-85 μmol/L)
eGFR	>90 mL/minute	>59 mL/minute
Calcium corrected	9.9 mg/dL (2.47 mmol/L)	8.82-10.42 mg/dL (2.20-2.60 mmol/L)
Glucose fasting	82.9 mg/dL (4.6 mmol/L)	64.9-108.1 mg/dL (3.6-6.0 mmol/L)
**Pituitary hormone panel**		
ACTH	17.3 pg/mL (3.8 pmol/L)	7.3-63.1 pg/mL (1.6-13.9 pmol/L)
9 Am cortisol	16.4 µg/dL (452 nmol/L)	4.8-19.5 µg/dL (133-537 nmol/L)
IGF-1	152 ng/mL (20 nmol/L)	61.2-221.8 ng/mL (8-29 nmol/L)
TSH	1.08 µIU/mL (1.08 mU/L)	0.5-5.5 µIU/mL (0.5-5.5 mU/L)
Free T4	1.43 ng/dL (18.4 pmol/L)	0.85-1.7 ng/dL (11.0-22.0 pmol/L)
Free T3	2.9 pg/mL (4.6 pmol/L)	2.0-4.2 pg/mL (3.1-6.4 pmol/L)
FSH	46 mIU/mL (46 IU/L)	Postmenopausal 31-153 mIU/mL (31-153 IU/L)
LH	22.7 mIU/mL (22.7 IU/L)	Postmenopausal 12-75 mIU/mL (12-75 IU/L)
Prolactin	10.3 ng/mL (10.3 μg/L)	4.2-29.6 ng/mL (4.2-29.6 μg/L)
Estradiol	15.3 pg/mL (56 pmol/L)	Postmenopausal <54.5 pg/mL (<200 pmol/L)

Abnormal value is shown in boldface. Values are shown as: conventional, (SI).

Abbreviations: ACTH, adrenocorticotropic hormone; eGFR, estimated glomerular filtration rate; free T3, free triiodothyronine; free T4, free thyroxine; FSH, follicular stimulating hormone; IGF-1, insulin like growth factor 1; LH, luteinizing hormone; TSH, thyroid stimulating hormone.

## Treatment

She was contacted by phone promptly and reported feeling well, with no symptoms of headaches, nausea, dizziness, or weakness, and noting tirzepatide as a new medication. She was advised to cease further desmopressin until polyuria developed and go to the emergency department for supervised sodium correction with her endocrinologist's plan of withholding desmopressin until polyuria, sodium monitoring every 4 to 6 hours, and corticosteroid replacement for safety.

On admission her serum sodium was 121 mEq/L [SI: 121 mmol/L]. She was not fluid restricted due to being asymptomatic, and polyuria was anticipated after stopping desmopressin. The sodium levels remained stable for 16 hours until polyuria began, then rose quickly to 131 mEq/L [SI: 131 mmol/L] in 3 hours. After taking 100 mcg oral desmopressin (26 hours post last dose), the sodium remained stable and desmopressin was administered once daily at 100 mcg, timed to the onset of polyuria.

## Outcome and follow-up

During her 4-day hospital stay, she remained asymptomatic with no neurological sequelae and discharged home requiring only a quarter of her previous desmopressin dose (100 mcg nightly). Wishing to continue tirzepatide for weight loss, she received additional guidance about “desmopressin escape,” and was advised to wait for polyuria before dosing.

Four weeks later, she underwent successful transsphenoidal RCC removal and her hydrocortisone replacement was discontinued due to normal postoperative cortisol levels.

## Discussion

This is the third reported case of severe hyponatremia with the use of tirzepatide [7, 8], and first case in a person with AVP-D. This new case is notable as it raises concern for those with AVP-D, given the increased risk for hyponatremia due to the presence of desmopressin.

GLP-1 RAs and dual GIP/GLP-1 RAs play a central role in addressing type 2 diabetes and obesity. Common side effects are gastrointestinal in nature [10]. Hyponatremia has been reported only recently with 2 severe cases occurring after short-term tirzepatide use. Shah et al describe a healthy 63-year-old woman who developed severe, symptomatic hyponatremia 4 days after starting tirzepatide [7]. Her clinical presentation included seizures, with a serum sodium of 122 mEq/L [SI: 122 mmol/L]. The second case report by Farhat et al documents a healthy 59-year-old man who developed severe, symptomatic hyponatremia with a serum sodium of 115 mEq/L [SI: 115 mmol/L], with an acute kidney injury due to dehydration after the sixth injection of tirzepatide [8]. Neither case had AVP-D.

A recent publication described 3 people with AVP-D who required significant reductions in their desmopressin regimen while taking GLP-1 RAs, after noting reductions in urine output [9]. In the first instance, a 70-year-old man taking semaglutide 1 mg weekly for 4 months showed a mild decline in sodium to 133 mEq/L [SI: 133 mmol/L], prompting a halving of oral desmopressin from 400 to 200 mcg daily. The remaining cases involved 2 women, using liraglutide and semaglutide, respectively, also reporting decreased thirst and urine output, necessitating major dose adjustments of desmopressin from 250 to 150 mcg daily and from 200 to 100 mcg daily, respectively. However, neither of these women experienced hyponatremia [9].

The effects of GLP-1 RAs on water balance are being explored, but evidence shows that they decrease thirst and urine output. In a study of 20 healthy volunteers, subcutaneous dulaglutide given at 1.5 mg for 3 weeks led to a median reduction in fluid intake (by 100 mL, *P* = .06) and 24-hour urine output (by 300 mL, *P* = .04), compared to placebo [11]. Similarly, in 34 participants with primary polydipsia, dulaglutide reduced fluid intake by 500 mL (*P* = .002) and urine output by 943 mL (*P* < .1) [12]. In the combined study of the 54 participants above, copeptin levels reduced by 12% (*P* = .047) without a change in serum or urine osmolality, renin, aldosterone, or cortisol, but did reduce 24-hour urinary sodium excretion (median 25 mmol/24 hours, *P* = .002) [13]. These results indicate that GLP-1 RAs reduce endogenous AVP levels, which is unexpected because decreased urine output typically suggests increased AVP secretion or action. The observed reduction in copeptin (and thus AVP) is contrary to Shah et al's proposal that tirzepatide-induced hyponatremia results from the syndrome of antidiuretic hormone secretion in their case report [7].

Authors Nakhleh et al propose mechanisms underlying the observed reduction in urine output: GLP-1 RAs decrease AVP levels resulting in reduced vasopressin V2 receptor-mediated sodium reabsorption in the thick ascending limb of the loop of Henle and reduced aquaporin 2 (AQP-2) expression in the apical membrane of collecting duct principal cells in the distal nephron, thereby diminishing osmotic permeability of collecting ducts [9]. This process promotes natriuresis and increases fluid delivery to the distal nephron, ultimately enhancing water excretion. Desmopressin enhances AQP-2–mediated water reabsorption, thereby enhancing water retention and increasing the risk of hyponatremia [9].

In considering the role of GIP and hyponatremia, GIP is predominantly metabolized in the kidney, but GIP receptors have not been detected in renal tissue [14]. In rat studies it has been shown that GIP infused intraportally leads to reduce renal perfusion, but the significance of this effect in humans is not known [15].

The relationship between weight loss, desmopressin dose, and hyponatremia risk is not clear. In adults, desmopressin doses are titrated to symptom control and are not weight-based [16]. Two observational studies of 134 and 459 adults with AVP-D, respectively, found a positive correlation of desmopressin dosage to body mass index [5, 17]. An observational study of 116 adults with craniopharyngioma-related obesity treated with GLP-1 RAs (exenatide, dulaglutide, liraglutide, and semaglutide) reported a mean weight change of 7.8 kg over 44.4 months. Reported adverse effects included increased hydrocortisone intake (24%), adrenal crises (12%) and “vasopressin decompensation” in 10%, with 30% hospitalized: these events were mostly triggered by infections. Vasopressin decompensation was defined as “changes in fluid balance with changes in natremia, requiring desmopressin adjustment or inpatient care,” with no further details given, and the majority of patients had panhypopituitarism, which complicates the clinical picture [18].

In this current case report, hyponatremia was attributed principally to the interaction between tirzepatide and desmopressin, supported by literature on GLP-1 RAs influencing water retention. Despite having a recurrent RCC, there was no evidence of cortisol deficiency at presentation. Cortisol deficiency may cause hyponatremia through AVP-dependent or independent mechanisms, such as decreased renal blood flow, glomerular filtration, and altered water permeability in distal tubules [19]. RCC causing pituitary stalk compression and increased AVP secretion is an extremely rare phenomenon, whereas AVP-D occurs in up to 67% of large RCCs [20]. Although telmisartan, which the patient was also taking, is rarely linked to hyponatremia, its involvement should be acknowledged [21, 22].

AVP-D guidelines offer limited advice on potential drug interactions that could intensify desmopressin's effects, such as certain antiepileptic drugs, because these combinations may increase renal sensitivity to desmopressin [23, 24]. Recently, we highlighted the risk of rapid and severe hyponatremia when desmopressin is combined with nonsteroidal anti-inflammatory drugs and opioids [6]. Numerous guidelines on AVP-D management recommend withholding or delaying a weekly dose of desmopressin known as “desmopressin escape” to allow polyuria [23-26], as this reduces the risk of out-of-hospital hyponatremia [27].

Heightened awareness and consistent vigilance are essential when prescribing tirzepatide in patients with AVP-D. Thorough education regarding “desmopressin escape,” appropriate reduction of desmopressin dosage, and maintaining a low threshold for serum sodium monitoring are advisable when treating individuals with AVP-D with tirzepatide.

## Learning points

- AVP-deficiency necessitating replacement with desmopressin is a serious medical condition that predisposes to hyponatremia.
- This is the third case of severe hyponatremia associated with tirzepatide and the first case in someone with AVP-D.
- Tirzepatide may increase renal water reabsorption and increase the risk of hyponatremia, particularly in those with AVP-D.
- Mitigation strategies such as practicing “desmopressin escape,” desmopressin dose reduction, and increased serum sodium monitoring are advisable when using tirzepatide in those with AVP-D.

## Contributors

C.C. was involved in the diagnosis and management of the patient, manuscript preparation, and revision and is accountable for all aspects of work.

## Data Availability

Original data generated and analyzed during this study are included in this published article.

## Abbreviations

AVP: arginine vasopressin
AVP-D: arginine vasopressin deficiency
GIP: glucose-dependent insulinotropic polypeptide
GLP-1 RA: glucagon-like peptide 1 receptor agonist
MRI: magnetic resonance imaging
RCC: Rathke cleft cyst
